# Thermodynamics of quantum systems with multiple conserved quantities

**DOI:** 10.1038/ncomms12049

**Published:** 2016-07-07

**Authors:** Yelena Guryanova, Sandu Popescu, Anthony J. Short, Ralph Silva, Paul Skrzypczyk

**Affiliations:** 1 H.H. Wills Physics Laboratory, University of Bristol, Tyndall Avenue, Bristol BS8 1TL, UK; 2 Département de Physique Théorique, Université de Genève, 1211 Genève, Switzerland

## Abstract

Recently, there has been much progress in understanding the thermodynamics of quantum systems, even for small individual systems. Most of this work has focused on the standard case where energy is the only conserved quantity. Here we consider a generalization of this work to deal with multiple conserved quantities. Each conserved quantity, which, importantly, need not commute with the rest, can be extracted and stored in its own battery. Unlike the standard case, in which the amount of extractable energy is constrained, here there is no limit on how much of any individual conserved quantity can be extracted. However, other conserved quantities must be supplied, and the second law constrains the combination of extractable quantities and the trade-offs between them. We present explicit protocols that allow us to perform arbitrarily good trade-offs and extract arbitrarily good combinations of conserved quantities from individual quantum systems.

## Abstract (web summary)

The second law of thermodynamics constrains how much of a conserved quantity, such as energy, can be extracted from a system in the form of work. Here, the authors generalize this law to quantum systems whose conserved quantities need not commute, showing that it is their combination to be constrained.

## Graphical abstract

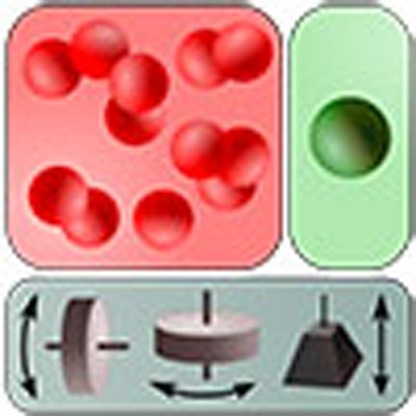

Thermodynamics is one of the most successful theories of nature that we have. Since its inception almost 200 years ago it has survived the transition from classical mechanics to relativistic and quantum mechanics, with its conceptual basis unchanged. The realm of thermodynamics has also been considerably extended, with recent years witnessing the extension of thermodynamics from dealing with macroscopic systems to individual quantum systems and black holes. From its earliest days, thermodynamics was also generalized to deal not only with energy but also with various conserved quantities, introducing grand canonical ensembles, chemical potentials and so on. Again, its conceptual basis remained unchanged.

In more detail, the idea of the grand canonical ensemble, where not only energy, but also the number of particles, is a conserved quantity, goes all the way back to Gibbs^1^. A milestone was the work in 1957 of Jaynes^2^,^3^, who, coming from a Bayesian perspective, suggested the generalization of thermodynamics to arbitrary conserved quantities through the principle of maximum entropy. The idea of the ‘generalized Gibbs ensemble' is by now commonly used in quantum statistical mechanics (see for example, refs 4, 5, 6, 7, 8). More recently, refs 9, 10 considered Landauer erasure given access to an ‘angular momentum bath' instead of a thermal bath, and demonstrated that information can be erased without an energy cost, provided the analogous cost is paid in angular momentum.

Very recently, there has been much renewed interest in the foundations of thermodynamics coming from the field of quantum information. On the one hand, the so-called ‘single-shot information theory', which was developed initially to study finite-size effects in quantum cryptography, has proven useful for studying finite-size effects and fluctuations in quantum thermodynamics, which has lead to the development of ‘single-shot quantum thermodynamics'^11^,^12^,^13^,^14^,^15^,^16^,^17^. On the other hand, inspired by so-called ‘resource theories', which have proved to be very powerful for studying quantum information tasks, such as the theory of entanglement^18^, purity^19^ or asymmetry^20^, the ‘resource theory of quantum thermodynamics'^21^ was developed that, in combination with single-shot framework, has generated a lot of interest and already shown itself to be a fruitful approach to quantum thermodynamics^22^,^23^,^24^,^25^,^26^,^27^,^28^,^29^,^30^,^31^,^32^,^33^,^34^,^35^,^36^. Our work proceeds along the lines of single-shot thermodynamics and resource theories, where a number of initial results concerning the thermodynamics of multiple conserved quantities were presented in refs 37, 38.

Here we present a generalized version of thermodynamics that deals with individual quantum systems and multiple—commuting or non-commuting—conserved quantities. What we will show is that unlike standard thermodynamics, where the second law constrains how much of the conserved quantity (energy) can be extracted from a non-equilibrium system, in the form of work, here there is no constraint on the amount of a single conserved quantity that can be extracted. In fact, we can extract as much of any individual conserved quantity as we like, if we supply an appropriate amount of other conserved quantities. What the second law constrains is the combination of conserved quantities that can be extracted—that is, the second law is seen to limit the trade-off of extractable quantities, which for the standard case of a single conserved quantity reduces to constraining how much can be extracted. At the same time, this generalized version of thermodynamics suggests that it may be worthwhile revisiting the basic concepts of the subject. Indeed, to understand the above phenomena we present an alternative viewpoint, which reinterprets some of the standard thermodynamics quantities, and is perhaps more natural and compelling. Closely related, independent work was performed by Lostaglio *et al*.^39^ and Halpern *et al*.^40^. In contrast to here, these works focus more on the nature of the thermal state itself when there are multiple conserved quantities.

## Results

### Overview

Here we consider the standard general framework of thermodynamics that consists of a thermal bath, an external system out of equilibrium with respect to the bath and a number of batteries, in which we will store various conserved quantities, which are extracted from the system and bath. In our case, following Jaynes^2^,^3^, we take the ‘thermal bath' to be simply a collection of particles, each described by a generalized thermal state


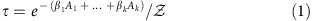


where *A*_*i*_ are various conserved quantities, *β*_*i*_ are the associated inverse temperatures and is the generalized partition function. Two things are important to note: first, the quantities *A*_*i*_ may or may not commute, and even when they commute they may or may not be functionally dependent on one another. Second, and most importantly, energy need not be one of the conserved quantities or indeed play any role. Since energy is the generator of time evolution, such a thermal bath may not arise naturally by thermal equilibration, but have to be created externally (for example, if the Hamiltonian is zero, then no evolution occurs). Yet, as we will see, the thermodynamic flavour of the theory remains.

The batteries are systems that can each store one of the conserved quantities *A*_*i*_. In our paper we will consider the batteries either explicitly or implicitly, as explained later. The system can be an individual quantum particle. Finally, the actions that we allow to be performed must conserve either exactly or on average each of the quantities *A*_*i*_, which is the content of the first law.

A central result of standard thermodynamics—the content of the second law—is that if we have access only to a single thermal bath, it is impossible to extract energy, in the ordered form of work, out of it, that is *W*:=Δ*E*^batt^≤0, where Δ*E*^batt^ is the change in the average energy of the battery. We show that, in our case, there is no limit on how much of any single conserved quantity *A*_*i*_ can be extracted, even though we have access only to a single generalized bath. More precisely, there is no limit on 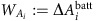. There is, however, a global limit.

In particular, to each conserved quantity we can associate an entropic quantity *β*_*i*_*A*_*i*_ (the entropic nature of this quantity will be explained later). We will show that these quantities can be almost perfectly interconverted for one another inside the bath. As a result, because of the first law (conservation of *A*_*i*_ between bath and battery) the only constraint on the 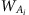 given just a thermal bath is that


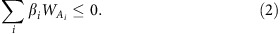


In standard thermodynamics the second law also says that if we have access to a system out of equilibrium with respect to the bath, then we can extract work, but we are limited by the change in free energy of the system, *W*≤−Δ*F*_s_. In our case, we define an entropic quantity, the ‘free entropy' of the system relative to the generalized bath, 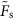,


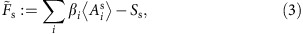


where *S*_s_ is the system entropy and show that


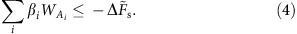


We will show, with a minimal number of assumptions, that we can implement any trade-offs between conserved quantities satisfying equation (2) using the bath, and extract any combination of 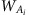 satisfying equation (4) from a system, up to an arbitrarily small deficit because of the finite nature of the protocols. In particular, if all the conserved quantities commute, we will give explicit protocols that works for both implicit and explicit batteries, assuming exact conservation of the *A*_*i*_. For more general non-commuting quantities, we will obtain the same results for implicit batteries, or explicit batteries with average conservation, but leave open the question of how to deal with strict conservation of non-commuting quantities when considering explicit battery systems.

### The generalized thermal state

Here we consider in more detail the generalized thermal state given in equation (1)^2^,^3^.

We begin by recalling that there are two ways to define the thermal state—by maximizing the von Neumann entropy, given appropriate constraints, or by minimizing the free energy. We start with the former. Consider a system in state *σ* with Hamiltonian *H* and average energy 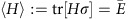. There are many states *σ* that have this particular average energy; the thermal state is the state that maximizes the entropy *S*(*σ*)=−tr(*σ* ln *σ*), subject to the average energy constraint. Solving the maximization problem we get 
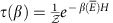
, where is the partition function and the inverse temperature *β* is implicitly determined by the average 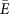.

In our framework we need the generalization of this idea to the case of multiple conserved quantities. In particular, we consider *k* quantities *A*_*i*_,*i*∈{1,⋯, *k*} and place no restrictions on the relations between them: they may or may not commute; when they commute they may or may not be functionally dependent on one another. An example of two commuting and functionally dependent quantities are the Hamiltonian *H* and angular momentum *L*, where *H*=*L*^2^/2*I*. In this case the average of one does not uniquely determine the average of the other; however, the range of admissible values is constrained, that is, such that 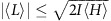. An example of two non-commuting conserved quantities is *L*_*x*_ and *L*_*y*_. The generalized thermal state *τ*(*β*_1_,…,*β*_*k*_) is then the state, which maximizes the entropy *S* subject to the constraint that the conserved quantities *A*_*i*_ have average value 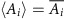. It is found to be

*Definition 1.* Generalized thermal state


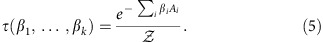


where, *β*_*i*_ is the inverse temperature conjugate to *A*_*i*_, and the generalized partition function is 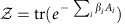.

Note that, in general, each *β*_*i*_ is a function of all of the averages 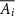. In the case that the *A*_*i*_ commute, the proof is a simple generalization of the standard proof. For non-commuting observables, the proof is more involved^41^.

The second way to define the thermal state (when only energy is conserved) is to fix the inverse temperature 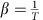 and ask for the density matrix that minimizes the free energy *F*(*ρ*)=〈*H*〉−*TS*(*ρ*). The state that solves this optimization has exactly the Gibbs form 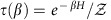. Since *β* is given, the average energy is now implicitly defined, in contrast to the case above, where the average energy was given and the inverse temperature derived.

The idea is to do the same in the case of multiple conserved quantities, and recover the generalized thermal state via a generalized free energy. However, in the standard definition of free energy the temperature is the constant multiplying the entropy. Since we have no notion of multiple entropies, we are not afforded a way of coupling all the inverse temperatures. This is easily overcome if instead of the free energy we define 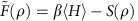, and it is trivial to generalize this quantity to the case of multiple conserved observables.

*Definition 2*. Free Entropy. The free entropy of a system *ρ* is


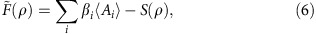


The free entropy is always defined with respect to a set of inverse temperatures *β*_*i*_. The generalized thermal state is then the state that minimizes 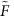 with fixed *β*_*i*_. For a complete proof of this fact, see the Supplementary Note 1.

### Conceptual viewpoint

As noted in the introduction, the effects presented in this paper suggest that it may be worthwhile to revisit the basic concepts of thermodynamics. A key aspect of this is the conceptual shift from the free energy to the free entropy.

First, we would like to emphasize that the change from the usual free energy to the free entropy is not a simple mathematical manipulation, but marks a fundamental conceptual difference. Indeed, in the standard approach to considering multiple conserved quantities, such as when considering the grand canonical ensemble, one introduces the chemical potential *μ* such that the free energy becomes


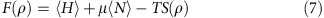


where *N* is the particle number operator. In this way, energy is singled out as the privileged quantity, with the chemical potential acting as the ‘exchange rate' between particle number and energy (and in the same way temperature acts as the exchange rate between entropy and energy). We argue that there is no reason to single out the energy, or any other quantity for that matter. In fact, it is possible to conceive of situations in which everything is degenerate in energy, and thus where energy plays absolutely no role. We are thus lead to introduce the free entropy, which naturally and uniquely treats all quantities on an equal footing.

A second argument for considering free entropy over the free energy is that the latter might give one incorrect intuition. Indeed, in the standard treatment, the free energy puts bounds on how much of the conserved quantity (energy) can be extracted, and one may be tempted to think that even when we have multiple conserved quantities, thermodynamics is about the bounds that constrain the extraction of individual quantities. However, as we will show, this is not the case, and there are no such bounds. The only limitation is on the trade-off between the conserved quantities, and this is precisely governed by the free entropy. It is only in the standard case of a single conserved quantity that one can choose to consider the free energy, or the free entropy, with both constraining the amount of work that can be extracted.

We also note that the thermal state is the state that minimizes the free energy only when the temperature is positive; if the temperature is negative the thermal state (at negative temperature) is instead the state that maximizes the free energy. On the other hand, for all temperatures (positive or negative), the thermal state always minimizes the free entropy.

Finally, we note that it is the difference in free entropy between *ρ*_s_ and *τ*_s_ that captures the thermodynamic usefulness of the system. This difference is exactly equal to the relative entropy between these two states, 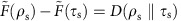, where 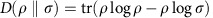. This is demonstrated explicitly in the Methods. This highlights the entropic nature of 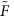.

Following on from the introduction of the free entropy, one can go a step further. Since the quantities *β*_*i*_〈*A*_*i*_〉 all appear alongside the entropy in the definition of the free entropy, it suggests that they might be thought of as ‘entropic quantities'. Note that this is true even in the standard case, where energy is the only conserved quantity; there one might think of *β*〈*H*〉 as an entropic quantity.

Importantly, the sum of these entropic quantities of the batteries is the object onto which the second law of thermodynamics applies. It says that the increase in this sum is constrained by the decrease in the free entropy of the system relative to the bath equation (4).

### The set-up

**Figure 1:**
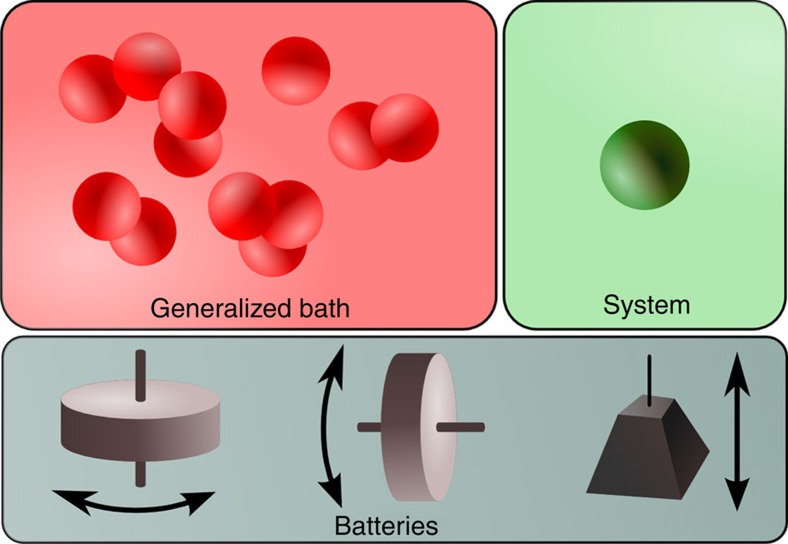
Schematic diagram of the set-up. We have a generalized bath, a collection of particles each in the generalized thermal state (1). We have a system that is out of equilibrium with respect to the generalized bath. Finally, we have the collection of batteries, one for each conserved quantity. In the example depicted, the three conserved quantities are the angular momentum in the *x* direction, angular momentum in the *z* direction and the energy, with the three corresponding batteries being turntables spinning around the *z* and *x* axes, respectively, and a weight. We can perform any interaction between the components as long as each of the conserved quantities is conserved (the first law).

The set-up is similar in spirit to that of previous lines of work^25^,^42^. We consider the interaction of generalized thermal baths with quantum systems and batteries (Fig. 1). There are a number of conserved quantities, *A*_1_ to *A*_*k*_, which may or may not commute or functionally depend on each other. The generalized thermal bath consists of an unbounded collection of systems, each of which is in a generalized thermal state as defined by equation (5). Any given protocol will involve only a finite set of systems in the bath, whose combined thermal state can be written as *τ*_b_(*β*_1_,…,*β*_*k*_). We also want to consider an additional quantum system *ρ*_s_ that is both initially uncorrelated from and out of equilibrium with respect to the generalized bath, that is, *ρ*_sb_=*ρ*_s_⊗*τ*_b_(*β*_1_,…,*β*_*k*_) and *ρ*_s_≠*τ*_s_(*β*_1_,…,*β*_*k*_). The main question we ask is how much of each of the conserved quantities can be extracted from the system (in conjunction with the bath, and stored in an associated battery).

In the interest of being clear, we proceed by concentrating on a scenario with only two conserved quantities, *A* and *B*, since this already captures the majority of the physics contained in the general case of *k* conserved quantities.

In order to talk about the extraction of the conserved quantities, there are two ways in which one can proceed: by either including battery systems implicitly, or explicitly, in the formalism. In the former case, one allows the global amount of each quantity stored in the system and bath to change, and defines the changes as the amount of ‘*A*-type work' and ‘*B*-type work' that have been extracted from (or done on) the global system. The idea is that because of global conservation laws, when the *A*_*i*_ of the system and bath changes, this change is compensated by a corresponding change to the external environment (the implicit battery).

In the latter case, one introduces explicit battery systems, which by definition only accept a single type of work (that is, an *A*-type battery and a *B*-type battery). Here by definition the amount of *A* stored in the *A*-type battery is the *A*-type work, and similarly for *B*. We enforce that the global amount of *A* and *B* stored in the system, bath and battery is constant, either strictly (the entire distribution is conserved) or on average.

In the main text we consider the case of implicit batteries. We do this since dealing with implicit batteries simplifies the considerations and allows us to focus on what is arguably the most important part of the protocols, namely the interaction between system and bath. Obviously, it is preferable to have the full protocol including batteries explicitly. In doing so, there are many subtleties, which also arise in the case of standard thermodynamics. In particular, we need to impose ‘no cheating' conditions that make sure that we do not make illegitimate use of batteries as sources of free entropy^25^,^42^,^43^. The danger stems from the fact that the batteries are systems out of equilibrium with respect to the bath. In Supplementary Note 2 we show how to include explicit batteries for a number of cases, as specified in Table 1.

More concretely, when considering implicit battery systems the class of allowed transformations consists of all global unitary transformations *U* on the system and bath. After such a transformation, the global state is 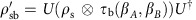 with the reduced state of the system and bath given by the reductions, 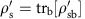 and 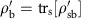, respectively. We define the *A*-type and *B*-type work to be


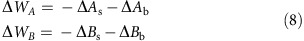


where 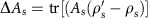, 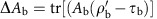 and analogously for Δ*B*_s_ and Δ*B*_b_. In addition, note that if our protocol involves multiple bath systems then 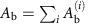, where 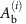 acts non-trivially only on bath system *i*, that is, *A*_b_ is the sum of the local *A* for each system (and analogously for *B*_b_). In equation (8) we are equating the average change of *A* and *B*, because of the unitary transformation, with the amount of *A*-type and *B*-type work that has been extracted from the system and bath. As such, our framework automatically incorporates the first law of thermodynamics for each of the conserved quantities.

Finally, an additional unrelated problem, but which often plays an important role, concerns the precise structure of the bath. In usual treatments, we may consider particles in the bath that have any energy-level spacing, such that their occupation probabilities can match any probabilities in the external system. This is used to construct efficient protocols. When considering other quantities than energy, we may be faced with quantities whose spectrum is fixed, such as angular momentum. In addition, extra constraints or relationships may exist between the different conserved quantities. This results in additional difficulties. To address these, and to remain as general as possible, we will consider baths with a minimal amount of accessible structure in terms of the eigenvalues of the conserved quantities.

### The second law

Of great interest to us is the particular form that the second law of thermodynamics takes in the present setting. In the classical thermodynamic setting, the second law states that if one only has access to a thermal bath, then no work can be extracted, and that the maximal amount of work that can be extracted from a non-equilibrium system interacting with a thermal bath is bounded by the change in its free energy.

In our framework of multiple conserved quantities, we will see that the second law constrains the different combinations of conserved quantities that can be extracted from the system. In particular, we will show below that in the present framework, the amount of *A*-type work and *B*-type work that can be extracted is constrained such that


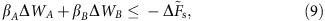


where 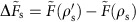. In the case where there is no system, or when the system is left in the same state, 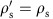, then 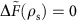, and we obtain as a corollary


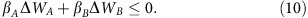


Equations (9) and (10) constitute the second law when one has multiple conserved quantities (with and without a system).

To prove the second law, equation (9), we will need to use two further formulae, as well as the first laws, equation (8). First, since we restrict to unitary transformations, the total entropy of the global system remains unchanged, 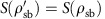, and from the fact that the system and bath are initially uncorrelated, along with sub-additivity, we have


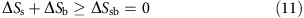


where 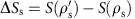, and analogously for Δ*S*_b_ and Δ*S*_sb_. Second, since the bath starts in the thermal state *τ*(*β*_*A*_, *β*_*B*_), which is a minimum of the free entropy (by definition), its free entropy cannot decrease during the protocol; thus,


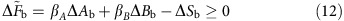


Now, eliminating all quantities on the bath, by substituting from the first laws, equation (8), and from equation (11), we finally arrive at


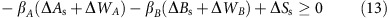


which, after re-arranging and identifying terms, is straightforwardly seen to be equation (9), as desired. Thus, the first law, in conjunction with the lack of initial correlations (and sub-additivity), and the extremality of the generalized thermal state imply in a direct manner that systems obey a second law of the form given. We note that the proof does not rely on any particular properties of *A* and *B*, which need not even commute.

At this point it is worth briefly returning to the issue of implicit versus explicit batteries. If explicit batteries are included, then the unitary operations have to be extended to act on the system, bath and explicit batteries. Crucially, equation (11), and as a consequence the second law, equation (9), can be shown to hold when we are careful to avoid cheating via batteries. Details are provided in Supplementary Note 2.

In the remaining we will study to what extent we can saturate equations (9) and (10), depending upon the properties of the conserved quantities (whether they commute or not), whether we consider implicit or explicit batteries and whether we consider strict or average conservation.

### Commuting observables

We will now specialize to the case of commuting observables, where we have access to joint eigenstates, and show how the second law can be saturated, both in terms of trading resources, and when extracting resources from a non-equilibrium system.

In order to remain as general as possible, we want to assume as little as possible about the structure of the generalized thermal bath. What we will require is that there exists a system in the bath (of which we can take arbitrarily many copies) with *d*≥3 states, |*a*_*i*_, *b*_*i*_〉, for *i*∈{0, 1, 2,…*d*−1}, which are the joint eigenstates of *A*_b_ and *B*_b_ such that *A*_b_|*a*_*i*_, *b*_*i*_〉=*a*_*i*_|*a*_*i*_, *b*_*i*_〉 and *B*_b_|*a*_*i*_, *b*_*i*_〉=*b*_*i*_|*a*_*i*_, *b*_*i*_〉. We then need only three requirements. First, that the three eigenvalues of each observable are distinct, *a*_0_≠*a*_1_≠*a*_2_ and *b*_0_≠*b*_1_≠*b*_2_ (note that one consequence of this is to rule out the case in which either *A* or *B* is proportional to the identity, and thus trivially cannot be changed). Second, that the observables should be sufficiently different. In particular, that


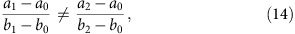


which amounts to saying that *A* and *B* should not be affinely related to each other, in which case they should not be thought of as different quantities. Third, that in the thermal state the joint eigenstates should not have the same populations. In particular, it must be that


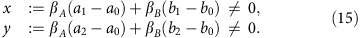


If both *x* and *y* simultaneously vanish, then all three states have the same populations, in which case the system looks maximally mixed in this subspace. When trading quantities inside the bath, this will be the only problematic case. However, when we come to processing non-equilibrium systems, we will require simultaneously *x*≠0 and *y*≠0 in order for the bath to have enough structure to allow us to approach reversibility. We will also see that, depending on how close to reversible we want to be, we will have to exclude a small set (non-dense and of measure zero) of joint values for *x* and *y*, which are rationally related, as will be explained later. Below we outline the main ideas, and present all the details in the Methods.

We start by considering the task of trading resources within the generalized bath. That is, we consider the situation where we only have access to a generalized bath (and no external system). We will show that we can perform a unitary transformation such that, first, its free entropy changes by an arbitrarily small amount,


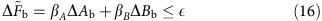


where we used that Δ*S*_b_=0 by definition for unitary transformations. Second, the change Δ*A*_b_ or Δ*B*_b_ can be made arbitrarily large, that is


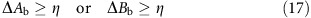


with the other appropriately constrained by equations (16); (generally having large magnitude but opposite sign). If the above two conditions can be satisfied, we say that we can exchange *A* for *B* in an essentially reversible manner.

To show that this is possible, we proceed in two steps. We provide an explicit protocol that exchanges a suitably chosen two-dimensional subspace within the bath, and calculate the change in 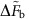, Δ*A*_b_ and Δ*B*_b_ that this produces. We then show that by repeating this protocol a sufficient number of times we achieve equations (16) and (17).

The explicit protocol takes the bath as *n* copies of *τ*(*β*_*A*_, *β*_*B*_), and a two dimensional subspace that consists of states that differ in population by Δ*q*, and differ in the number of systems in the state |*a*_1_, *b*_1_〉 by Δ*n*_1_. Then, as we show in the Methods, by interchanging the population of two such states, we can achieve


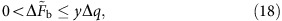



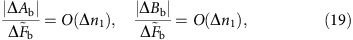


where the sign of Δ*A*_b_ or Δ*B*_b_ can be chosen arbitrarily, with the other quantity generally having the opposite sign, in accordance with equation (16). Hence, 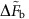 can be made as small as desired by making Δ*q* arbitrarily small, while independently the relative change 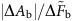 can be made as large as desired by increasing Δ*n*_1_.

Finally, by repeating the above protocol a sufficient number of times, one can trade arbitrary amounts of the conserved quantities from a generalized bath by sacrificing an arbitrarily small amount of free entropy. In particular, to achieve 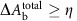 with 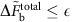, one can perform the protocol above (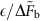) times, with 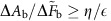.

We now move onto the task of extracting resources from a single quantum system. In the above we showed that arbitrarily good interconversions can be enacted, given access only to a generalized bath. We now move on to the scenario of having a quantum system out of equilibrium with respect to the bath. Our goal is to show that we can saturate the second law given by equation (9) arbitrarily well—that is, that we can extract conserved quantities from a non-equilibrium system such that *β*_*A*_Δ*W*_*A*_+*β*_*B*_Δ*W*_*B*_ is as close as desired to the system's decrease in free entropy.

Let us consider that we have a state *ρ*_s_, which in terms of its eigenstates and eigenvalues is given by *ρ*_s_=∑_*i*_*p*_*i*_|*ψ*_*i*_〉〈*ψ*_*i*_|, and by convention we take the eigenvalues to be ordered, *p*_*n*+1_≤*p*_*n*_. In general, the eigenbasis of the state will not coincide with the joint eigenbasis of the conserved quantities *A* and *B*. The first step is to pre-process the system, to bring it to a diagonal form in this basis. As we show in the Methods, it is always possible to do so, without even utilizing the bath, such that 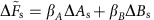, that is, in a fully reversible way that saturates equation (9). Note that in the case of explicit battery systems this pre-processing step is more complicated, nevertheless, the ideal pre-processing can still be arbitrarily well approximated, as shown in full details in Supplementary Note 3.

Now, having bought the system to diagonal form, we want to consider a protocol that moves a small population *δp* between two eigenstates, which have populations *p*_0_ and *p*_1_, respectively. We can implement such changes by finding two levels in the bath whose ratio of populations are close to *p*_0_/*p*_1_, and then swapping the 2D subspaces of the system and bath. Therefore, to carry out any such transformation requires a finely spaced set of different population ratios in the bath.

In the Methods we show that, except for a non-dense and measure zero set of *x* and *y* (as defined in equation (15)), we can find two levels in the bath whose ratio of populations is sufficiently close to *p*_0_/*p*_1_, such that after applying the swap operation between the appropriate two-dimensional subspaces, we achieve


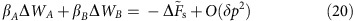


that is, up to a correction of order *O*(*δp*^2^), the combination of conserved quantities extracted, which themselves are order *O*(*δp*), matches the change in free entropy of the system. Thus, by composing *O*(1/*δp*) of such transformations, we can implement a protocol that transforms *ρ*_s_→*τ*_s_(*β*_*A*_, *β*_*B*_), whereby in each stage the population changes between two states by order *O*(*δp*), and such that


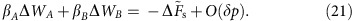


Therefore, by taking *δp* sufficiently small we can approach the reversible regime, whereby the change in free entropy of the system matches the dimensionless combination of conserved quantities extracted. Combining this protocol with the protocol from the previous section, involving only the generalized bath and the batteries, we can obtain any combination of extracted conserved quantities.

Finally, note that the same protocol can also be used to perform efficient transformations between any two system states (where the final state is full rank), and not just to the thermal state. Our protocol also immediately gives an asymptotic protocol for the interconversion of states with no average work cost: the rate at which one can transform *ρ*^⊗*n*^ into *σ*^⊗*nR*^ is given by


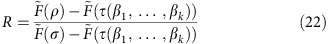


Here one can simply run the protocol ‘forward' individually on *n* copies of *ρ* in order to obtain in the batteries 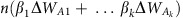, and create *τ*(*β*_1_,…,*β*_*k*_)^⊗*n*^. Then, on *nR* copies run the protocol ‘backwards' to create *σ*^⊗*nR*^, having returned each battery so that finally it contains the same amount its associated quantity that it initially contained (on average).

In Supplementary Note 3 we show how these results extend to the case of explicit batteries with either strict or average conservation. We also show in Supplementary Note 4 how the protocol can be made robust to experimental imperfections—that is, without assuming precise knowledge of *β*_*A*_ or *β*_*B*_.

### Non-commuting observables

In this section we will show that when considering implicit batteries the results obtained in the previous section can easily be modified to also work for non-commuting observables. This also extends to explicit batteries with average conservation laws when the batteries have continuous spectrum. However, the same protocols do not obviously generalize to the case of explicit batteries with strict conservation.

Whereas previously by virtue of the commutativity of the observables we could find a joint eigenbasis |*a*_*i*_,*b*_*i*_〉 that was used in our explicit protocols, that is no longer the case for non-commuting observables. Nevertheless, the generalized thermal state is diagonal in the eigenbasis of *β*_*A*_*A*+*β*_*B*_*B*,


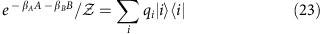


Although to each eigenstate we can no longer associate an eigenvalue for *A* or *B*, we can still associate an average value,


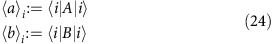


The main point is that all of our previous results hold if instead of joint eigenstates |*a*_*i*_, *b*_*i*_〉, with eigenvalues *a*_*i*_ and *b*_*i*_, we use the eigenstate |*i*〉 with average values 〈*a*〉_*i*_ and 〈*b*〉_*i*_ throughout.

The only subtlety that arises is the structure we need from the bath. We still only need to use three distinct eigenstates, |0〉, |1〉 and |2〉; however, now the necessary structure relates to the average value of the conserved quantities in the eigenstates, First, it must be that


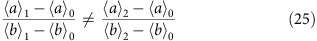


otherwise, at the level of the average values, the observables appear affinely related and therefore cannot be sufficiently distinguished to allow for trade-offs. Furthermore, we still need to be able to find eigenstates in the bath that differ in population, just as before. We can define the analogous quantities 〈*x*〉 and 〈*y*〉, and if they do not simultaneously vanish then the bath will not be maximally mixed in the subspace. Finally, in order to extract resources from systems out of equilibrium with respect to the generalized bath, there must be sufficient structure such that any ratio of populations can be approximated well enough. Again, in complete analogy to the above, if (〈*x*〉/〈*y*〉) is irrational, then we have sufficient structure. If on the other hand (〈*x*〉/〈*y*〉) is rational, we will again have to exclude a small set of values of 〈*x*〉 and 〈*y*〉 (non-dense, of zero measure), for which our results will not hold.

## Discussion

In this work we have studied a generalization of thermodynamics where there are multiple conserved quantities, where energy may not even be part of the story. We have been interested in what form the second law takes, and showed that it is no longer about restrictions on individual extractable quantities, but rather about the allowed ways that the conserved quantities can be traded-off for one another. Indeed, we found that we can extract as much of any individual conserved quantity as desired, as long as the other conserved quantities are appropriately consumed in the process, with the second law dictating how much of the others are necessarily consumed. In particular, we were led to introduce a dimensionless generalization of the free energy, which we termed the free entropy, that is the central quantity appearing in the second law and dictating the allowed trade-offs. Moreover, given access to any quantum system out of equilibrium with respect to the generalized bath, we showed that its free entropy change bounds the combination of conserved quantities that can be extracted.

Our results hold both for commuting and non-commuting observables, and with the desire to remain as general as possible we made only very mild assumptions about the bath. Indeed, we assumed very little about the relationship between the conserved quantities or their individual structure. The one case that remains open for future research is the case of non-commuting observables, with explicit batteries and strict conservation of the conserved quantities. Although the protocols presented for saturating the second law do not appear to generalize to this case, we do not know whether entirely different constructions will be able to achieve this goal.

## Methods

### Relation between free entropy and relative entropy

Here we show that the free entropy difference between any state *ρ*_s_ and the generalized thermal state 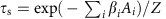 is equal to the relative entropy difference between these two states. First, note that the free entropy of the thermal state is


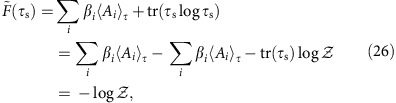


where we recall that 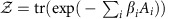 is the generalized partition function. Then, it follows that


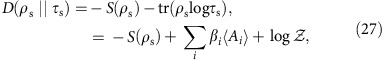


which demonstrates the claim. Note that this result is completely analogous to the case of standard thermodynamics^44^.

### Trading resources

Consider the situation where we only have access to a generalized bath (and no external system). We will show that we can perform a unitary transformation such that: (i) the free entropy 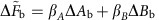 (since Δ*S*_b_=0 by definition) changes by an arbitrarily small amount. (ii) The changes |Δ*A*_b_| and |Δ*B*_b_| can be made arbitrarily large.

Consider that we have a *n* copies of 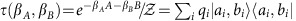. It will be convenient to label states by the number of systems found in a given eigenstate, which we shall refer to as the occupation. We denote by |**n**, *α*〉≡|*n*_0_, *n*_1_,…, *n*_*d*−1_, *α*〉=*P*_*α*_|*a*_0_, *b*_0_〉^⊗*n*_0_^|*a*_1_, *b*_1_〉^⊗*n*_1_^⋯|*a*_*d*−1_, *b*_*d*−1_〉^⊗*n*_*d*−1_^, where *P*_*α*_ is a permutation operator, permuting the bath systems, labelled by *α*, and *n*_0_+*n*_1_+…+*n*_*d*−1_=*n*. Now, we will consider only two states from the *d*^*n*^ which are available, corresponding to


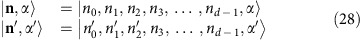


that is, such that only the occupations of the first three levels differ between these states. As such, we have the constraint that 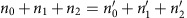. The key step in our protocol is to apply a swap operation between these two states, while leaving all other states unchanged. We assume the bath is sufficiently large so that after this step any modified systems from the bath can be discarded, and any further operations act on fresh bath systems. A direct calculation shows that the change in the average value of each quantity of interest is


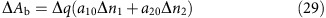



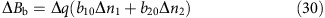



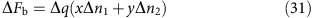


where Δ*n*_*k*_=(*n*′_*k*_−*n*_*k*_), *a*_*k*0_=(*a*_*k*_−*a*_0_), *b*_*k*0_=(*b*_*k*_−*b*_0_), *x* and *y* are as defined in equation (15), and


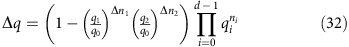


is the difference in populations between the two states. Given *y*≠0, we can rewrite equation (31) as


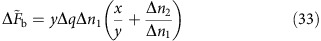


Now, for arbitrary Δ*n*_1_, we can find an integer *m* such that *m*/Δ*n*_1_<*x*/*y*≤(*m*+1)/Δ*n*_1_. Setting Δ*n*_2_=−*m* in equation (33), we obtain


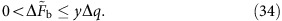


Hence, 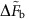 can be made as small as desired by making Δ*q* arbitrarily small (which can be achieved by increasing *n*_0_). Note that it is crucial that 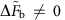. This is because the thermal state is the unique state that minimizes 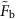. Thus, 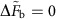 implies that the bath is left completely unchanged, which in turn implies that Δ*A*_b_=Δ*B*_b_=0; hence, the desired transformation cannot take place. On the other hand, we find that the relative change in the conserved quantities 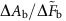 and 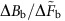 are


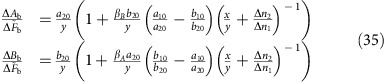


In both cases, the final term satisfies (*x*/*y*+Δ*n*_2_/Δ*n*_1_)^−1^≥Δ*n*_1_ and can, hence, be made as large as desired by increasing Δ*n*_1_. This means that the magnitude of 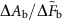 and 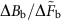 will become arbitrarily large. The sign of Δ*A*_b_ will depend on the other constants, but can be modified if desired by choosing *m* such that (*m*−1)/Δ*n*_1_≤*x*/*y*<*m*/Δ*n*_1_ above. Note also that if *y*=0 but *x*≠0 we can construct an equivalent proof with the roles of *x* and *y* swapped.

Finally, by repeating the above protocol a sufficient number of times, one can trade arbitrary amounts of the conserved quantities from a generalized bath by sacrificing an arbitrarily small amount of free entropy. In particular, to achieve 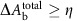 with 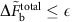, one can perform the protocol above (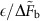) times, with 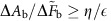.

There are a number of important aspects of the above protocol: first, it relies on a minimal amount of structure in the observables *A* and *B* and the bath: it requires that there exist many copies of a bath system with a three-dimensional subspace where the action of the operators are not trivially related (by a shift and rescaling), and that the state is not maximally mixed in this subspace. Moreover, each bath system is taken to be identical, with no additional parameters necessary (that is, we do not require a family of different *A*_b_′s and *B*_b_′s, similar to the families of Hamiltonians considered in ref. 25). Second, this protocol is a only a proof-of-principle demonstration that trade-offs can be enacted. No attention was paid to the number of generalized thermal states necessary. If one cared about minimizing the resources utilized, then the above protocol would not be used, and more efficient ones would be sought. Finally, the above analysis generalizes beyond two conserved quantities to the general case of *k* quantities. In this case, equation (14) must hold pairwise for all quantities.

### Extracting resources from a single quantum system

Here we consider the scenario involving a quantum system out of equilibrium with respect to the bath. We will show that the second law given by equation (9) can be saturated arbitrarily well—that is, we can extract conserved quantities from a non-equilibrium system such that *β*_*A*_Δ*W*_*A*_+*β*_*B*_Δ*W*_*B*_ is as close as desired to the system's decrease in free entropy.

Let us consider that we have a state *ρ*_s_, which in terms of its eigenstates and eigenvalues, is given by 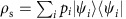, and by convention we take the eigenvalues to be ordered, *p*_*n*+1_≤*p*_*n*_. In general, the eigenbasis of the state will not coincide with the joint eigenbasis of the conserved quantities *A* and *B*. The first step is to pre-process the system to bring it to a diagonal form in this basis. To do so we will not interact with the bath, but simply apply the unitary


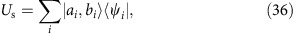


on the system such that 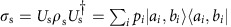. Owing to the first laws, equation (8), we have Δ*W*_*A*_=−Δ*A*_s_ and Δ*W*_*B*_=−Δ*B*_s_. Moreover, since the entropy of the system did not change, 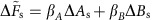, and we have immediately


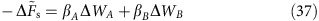


that is, in this state, unsurprisingly, we have a change in the batteries that coincides with the free entropy change of the system, and saturates equation (9). Note that in the case of explicit battery systems, the transformation in equation (36) cannot be perfectly performed at the level of the system, since [*U*_s_, *A*_s_]≠0 and [*U*_s_, *B*_s_]≠0. Nevertheless, one can implement a joint unitary on the system and batteries such that at the level of the system the transformation *U*_s_ can be approximated arbitrarily well as long as the battery systems are in appropriate states, similarly to the case of standard quantum thermodynamics^42^,^43^. Full details can be found in Supplementary Note 3.

Now, having brought the system to a diagonal form, we want to consider a transformation 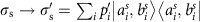 in which only two levels of the system change their populations by a small amount. Note that here we denote the eigenvalues of *A*_s_ and *B*_s_ by 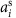 and 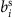 to differentiate them from the eigenvalues of *A*_b_ and *B*_b_, which we will similarly denote by 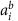 and 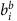. Namely, we would like to perform 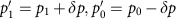. We can implement such changes by finding two levels in the bath whose ratio of populations is close to *p*_0_/*p*_1_ and then swapping the two-dimensional subspaces of the system and bath. To carry out any such transformation requires a finely spaced set of different population ratios in the bath. Let us now consider whether the simple bath systems we have considered so far can provide such possibilities. As before, we bring in a collection of *n* generalized thermal states, *τ*(*β*_*A*_,*β*_*B*_) and consider only the two states in equation (28), |**n**, *α*〉 and |**n**′, *α*′〉. We shall denote the populations of these states by *q*_**n**_ and *q*_**n**′_, respectively. The global unitary transformation we will apply is the swap operator between the two-dimensional subspaces of the system and bath, and the identity everywhere else. That is, the operation that performs


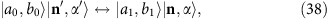


while leaving all other states unchanged. By performing this transformation, the population that is shifted between the states of the system, which coincides with the population that is shifted between the states of the bath, is *δp*=(*p*_0_*q*_**n**′_−*p*_1_*q*_**n**_). Now, the changes in the conserved quantities of the system and bath are found to be


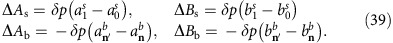


The change in the entropy of the system and the bath are also found to be


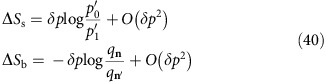


where we use 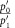 rather than 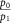 since we can always take 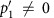. We want to achieve Δ*S*_s_+Δ*S*_b_=*O*(*δp*^2^), which requires that 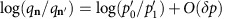. To see when this condition is satisfied, we first use the explicit form of the probabilities, which show that *q*_**n**_/*q*_**n**′_=exp(−*x*Δ*n*_1_−*y*Δ*n*_2_). Hence, we require 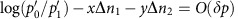. Since 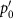 and 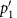 are arbitrary, our requirement is that *x*Δ*n*_1_+*y*Δ*n*_2_ should be able to come within *O*(*δp*) of any number. As we can rescale Δ*n*_1_ and Δ*n*_2_ by an arbitrary integer, it is sufficient to obtain 0<*x*Δ*n*_1_+*y*Δ*n*_2_≤*O*(*δp*). If *x* and *y* are not rationally related, this is always possible. However, if *x*/*y* is rational, and given in reduced form by *u*/*v* (where *u* and *v* are co-prime integers), then we need


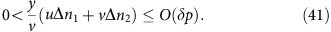


From number theory, one can always find Δ*n*_1_ and Δ*n*_2_ such that the term in brackets is 1 (or −1); hence, we need |*y*/*v*|≤*O*(*δp*). For a fixed desired accuracy 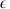 (of order *O*(*δp*)), and fixed *y*, this rules out a finite number of *x* values for which *x*/*y* is a rational with a small denominator. Extending this to the *x-y* plane, we find that we can achieve the desired accuracy everywhere except for a non-dense set of measure zero.

Returning to the entropies, with the above in place, Δ*S*_s_+Δ*S*_b_=*O*(*δp*^2^), that is, the system and bath remain essentially uncorrelated after the transformation. Finally, we use once again the fact that the generalized thermal state is a minimum of the free entropy. This implies that the changes in population, of order *O*(*δp*), change the free entropy only to second order, 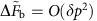. Putting everything together we have


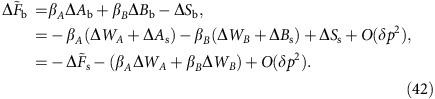


Thus, since the left-hand side is *O*(*δp*^2^), it must be that


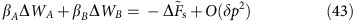


that is, up to a correction of order *O*(*δp*^2^), the combination of conserved quantities extracted, which themselves are order *O*(*δp*), matches the change in free entropy of the system. Thus, by composing *O*(1/*δp*) of such transformations, we can implement a protocol that transforms *ρ*_s_→*τ*_s_(*β*_*A*_,*β*_*B*_), whereby in each stage the population changes between two states by order *O*(*δp*), and such that


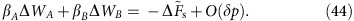


Therefore, by taking *δp* sufficiently small we can approach the reversible regime, whereby the change in free entropy of the system matches the dimensionless combination of conserved quantities extracted. Combining this protocol with the protocol from the previous section, involving only the generalized bath and the batteries, we can obtain any combination of extracted conserved quantities.

It is important to stress that our assumptions only changed by a small amount relative to the previous section. In particular, we need a minimal amount of extra structure in the bath, such that it is useful to process arbitrary individual systems out of equilibrium. Furthermore, as in the previous case the protocol presented generalizes in a straightforward manner to the case of *k* mutually commuting conserved quantities *A*_1_,... *A*_*k*_.

Finally, note that the same protocol can also be used to perform efficient transformations between any two system states (where the final state is full rank), and not just to the thermal state. Our protocol also immediately gives an asymptotic protocol for the interconversion of states with no average work cost: the rate at which one can transform *ρ*^⊗*n*^ into *σ*^⊗*nR*^ is given by


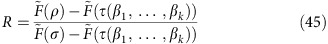


Here one can simply run the protocol ‘forward' individually on *n* copies of *ρ* in order to obtain in the batteries 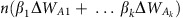 and create *τ*(*β*_1_,…,*β*_*k*_)^⊗*n*^. Then, on *nR* copies run the protocol ‘backwards' to create *σ*^⊗*nR*^, having returned each battery so that finally it contains the same amount its associated quantity that it initially contained (on average).

In Supplementary Note 4 we show how these results extend to the case of explicit batteries with either strict or average conservation. In Supplementary Note 4 we also show how the protocol can be made robust to experimental imperfections—that is, without assuming precise knowledge of *β*_*A*_ or *β*_*B*_.

### Data availability statement

Data sharing not applicable to this article as no datasets were generated or analysed during the current study.

## Additional information

**How to cite this article:** Guryanova, Y. *et al*. Thermodynamics of quantum systems with multiple conserved quantities. *Nat. Commun.* 7:12049 doi: 10.1038/ncomms12049 (2016).

## Supplementary Material

Supplementary Information Supplementary Figures 1-3, Supplementary Notes 1-4 and Supplementary References.

## Figures and Tables

**Table 1:** Summary of the results contained in the paper.

	Commuting	Non-commuting
*Implicit batteries*		
Second law	✓	✓
Protocol	✓	✓
		
*Explicit batteries (strict conservation*)		
Second law	✓	✓
Protocol	✓	?
		
*Explicit batteries (ave. conservation)*		
Second law	✓	✓
Protocol	✓	✓*

The second law equations (9) holds in all instances.

^*^Designates that the result holds only for explicit batteries with continuous spectra.
