# Supplementary material for: Thermodynamics of quantum systems with multiple conserved quantities
Source: Nat Commun. 2016 Jul 7;7:12049. doi: 10.1038/ncomms12049 (PMC4941046; doi:10.1038/ncomms12049)
Supplement: Supplementary Information — Supplementary Figures 1-3, Supplementary Notes 1-4 and Supplementary References. [file ncomms12049-s1.pdf]

SUPPLEMENTARY FIGURES

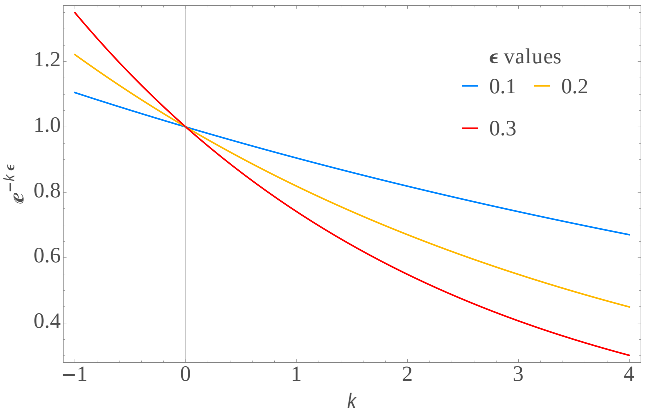

Supplementary Figure 1. A graph of $\frac{q_{\mathbf{n}'}}{q_{\mathbf{n}}} = e^{-k\epsilon}$ for different values of ϵ. As ϵ gets smaller, the values the function can attain for integer k become closer together (y-axis intercepts), until they are sufficiently dense to reproduce any real number.

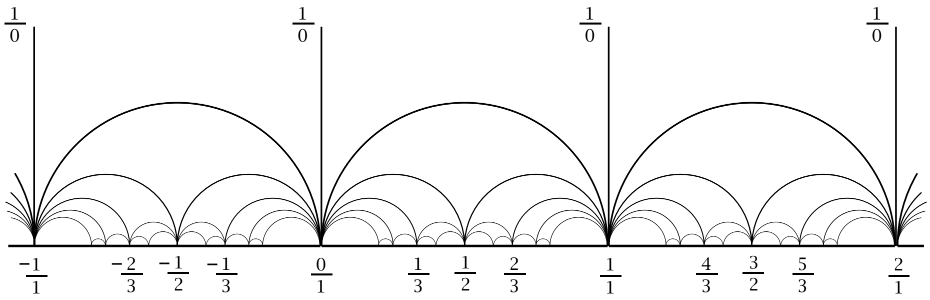

Supplementary Figure 2. The Farey sequence of order 5. Image credit [1].

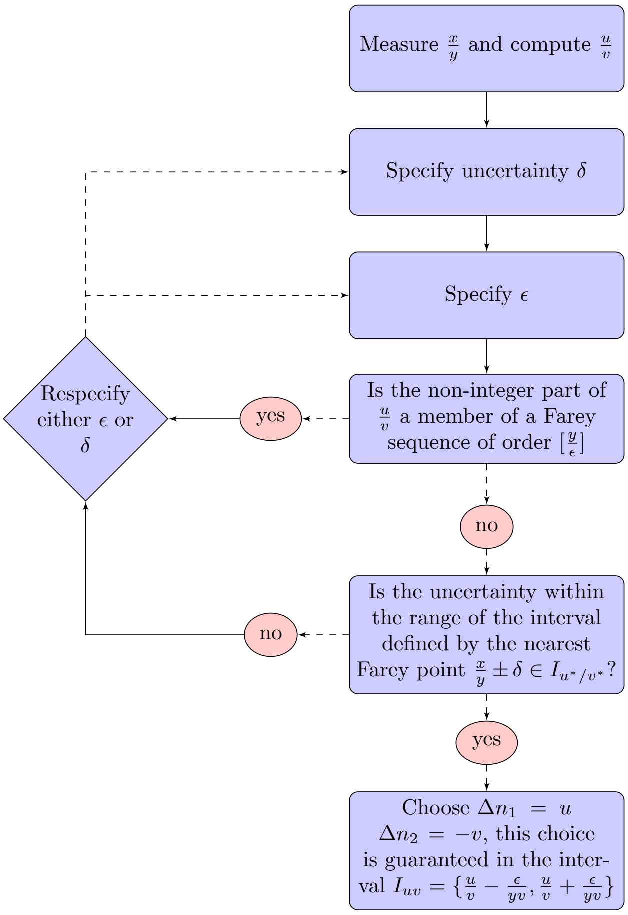

Supplementary Figure 3. Flow chart showing a robust protocol for work extraction in the presence of measurement uncertainty.

SUPPLEMENTARY NOTE 1

The generalised thermal state

In this section we prove that the generalised thermal state, in our framework of multiple conserved observables, can be obtained by either minimising the free entropy or by maximising the von Neumann entropy.

Minimising the free entropy is relatively simple, regardless of the relationship between the observables. We use the definition of free entropy from the main paper (Eq. 3) with n conserved observables labelled by A_i, $i \in \{1, \cdots, n\}$,

$$\tilde{F}(\rho) = \sum_i \beta_i \langle A_i \rangle - S(\rho). \tag{1}$$

where β_i are the inverse temperatures corresponding to each observable, $\langle A_i \rangle = \mathrm{tr}[A_i \rho]$ are the averages of each conserved quantity, and $S(\rho) = -\mathrm{tr}[\rho \log \rho]$ is the von Neumann entropy. We relabel the linear combination of observables as a single operator $R = \sum_i \beta_i A_i$ so that

$$\tilde{F}(\rho) = \mathrm{tr}[R\rho] - S(\rho). \tag{2}$$

We first note that the state which minimizes this function must be diagonal in the eigenbasis of R (If it was not, one could simply de-cohere the state in this basis, resulting in a state with an identical value for $\mathrm{tr}[R\rho]$ but higher entropy). If the occupation probability of the eigenstate with eigenvalue R_i is denoted by p_i, one can perform Lagrangian optimization to extremise

$$\mathcal{L}(\rho) = \sum_i R_i p_i + \sum_i p_i \log p_i + \lambda(\sum_i p_i - 1) \tag{3}$$

where λ is a Lagrange multiplier to obtain

$$\rho = \frac{e^{-R}}{\mathcal{Z}} = \frac{e^{-\sum_i \beta_i A_i}}{\mathcal{Z}} \tag{4}$$

where the partition function is $\mathcal{Z} = \mathrm{tr}[e^{-\sum_i \beta_i A_i}]$. Our aim is now to show that we arrive at the same form of solution if we maximise the von Neumann entropy subject to the averages of the conserved observables being fixed. There are two cases to consider here, depending on whether the observables A_i commute with each other or not.

Commuting observables

In this case, we wish to maximise the entropy subject to the constraints that all n observables commute with one another i.e $[A_i, A_j] = 0, \quad \forall i, j \in \{1, \cdots, n\}$ and that each observable has some fixed average value on the system $\overline{A}_i$.
Since all of the observables commute, there exists a common eigenbasis, and the state that maximizes the entropy will necessarily be diagonal in this basis. (Otherwise, as above, one could de-cohere the state in the common eigenbasis, resulting in a state with the identical averages $\overline{A}_i$ but higher entropy).
The problem can be expressed as a Lagrangian optimization subject to the constraints that the state is normalised and the average observable quantities are constant

$$\begin{aligned} \mathcal{S}(\rho) &= -\mathrm{tr}[\rho \log \rho] - \sum_i \beta_i (\mathrm{tr}[\rho A_i] - \overline{A}_i) + \lambda(\sum_i p_i - 1) \\ &= -\sum_j p_j \log p_j - \sum_i \beta_i (\sum_j p_j m^i_j - \overline{A}_i) + \lambda(\sum_j p_j - 1) \end{aligned} \tag{5}$$

where we interpret λ and β_i as the Lagrange multipliers and m^i_j is the j-th eigenvalue of the i-th observable. By solving $\frac{\partial S}{\partial p_i} = 0$ we find the solution is

$$\rho = \frac{e^{-\sum_i \beta_i A_i}}{\mathcal{Z}}, \tag{6}$$

Non-commuting observables

For the case of non-commuting observables we will have to be more careful as we can no longer diagonalise them in the same basis.
First we imagine that the average of the conserved observables are given to us, i.e. we have the numbers $\overline{A}_i$ for $i \in \{1, \cdots, n\}$. Now we consider a state of the form

$$\rho = \frac{e^{-\sum_i \beta_i A_i}}{\mathcal{Z}}. \tag{7}$$

Since we know the operators A_i we can compute their averages on the state ρ as functions of the inverse temperatures β_i

$$A_i(\rho) = \mathrm{tr}[\rho A_i] = f_i(\boldsymbol{\beta}) \qquad \forall i \tag{8}$$

where $\boldsymbol{\beta}$ is the vector of inverse temperatures $(\beta_1, \cdots, \beta_n)$. For the average quantities that we have been given, we now solve for the β_i

$$f_i(\boldsymbol{\beta}) = \overline{A}_i \qquad\qquad \forall i \tag{9}$$

The fact there there always exist solutions is non-trivial. In particular it implies that for any given set of average values $\{\overline{A}\}_i$ (here we implicitly assume compatible average values), there exist corresponding temperatures β_i such that a state of the form (7) pertains to these averages. This result was first proved by Jaynes [2] and subsequently via a different method by Kai [3]. Solving these equations, we find the particular solutions β_i^*. We now define a new function $\tilde{F}^*$ which acts on density operators

$$\tilde{F}^*(\mu) = \sum_i \beta_i^* \langle A_i \rangle_\mu - S(\mu) \tag{10}$$

Next, consider a density operator γ with the properties that it has average values $\langle A_i \rangle_\gamma$ equal to $\overline{A}_i$ and that it also maximises the entropy $S(\gamma)$. Then $\tilde{F}^*(\gamma)$ is

$$\begin{aligned}\tilde{F}^*(\gamma) &= \sum_i \beta_i^* \langle A_i \rangle_\gamma - S(\gamma) \\ &= \sum_i \beta_i^* \overline{A}_i - S(\gamma) \end{aligned} \tag{11}$$

We now consider the unique density operator σ that minimises $\tilde{F}^*$. It can be obtained via the method in the first section and is simply

$$\sigma = \frac{e^{-\sum_i \beta_i^* A_i}}{\mathcal{Z}} \tag{12}$$

However, from Eqs. (8)) and (9) we observe that the state with inverse temperatures β_i^* and the form given in Eq. (12) has averages $\overline{A}_i$ (since the non-trivial solutions to a linear system are unique), which implies

$$\tilde{F}^*(\sigma) = \sum_i \beta_i^* \, \overline{A}_i - S(\sigma). \tag{13}$$

Since $\tilde{F}^*(\rho)$ is at a minimum, it implies that $S(\sigma)$ is maximum, thus

$$\tilde{F}^*(\sigma) = \tilde{F}^*(\gamma) \quad \implies \quad \sigma = \gamma \tag{14}$$

and we see that the state which maximises the entropy and has averages $\{\overline{A}_i\}_i$ is indeed the generalised thermal state.

SUPPLEMENTARY NOTE 2

Explicit batteries: allowed operations and the second law

In the main text we presented the proof of our claim for the case that the work storage systems (the batteries) were implicit. Here we present an extended framework in which the batteries are explicit.

Our setup is much the same as in the main text: we have a bath b, system s and in addition, two batteries, which we call weights w_A and w_B. We model the battery systems as weights in the most general sense, whose value for each observable is given by a position observable (for energy, the height of the weight corresponds to the stored energy, but note that these weights need not be gravitational). If the observable has a discrete spacing such as angular momentum, then the weight may be a ladder with discrete spacing, but otherwise we take it to be continuous.

The value of observable A on the weight is proportional to the position operator $A_{w_A} = c_a \hat{x}_a$, where c_a is a constant of appropriate units in order to recover the correct dimensions for the quantity A, and we define the work by $\Delta W_A = \Delta A_{w_A}$. At this stage it is also useful to define the translation operator

$$\Gamma_{w_A}^{\epsilon} = \exp(-i\epsilon \hat{p}_a) \tag{15}$$

where ϵ reflects the amount of translation of the weight and $\hat{p}_a$ corresponds to the operator canonically conjugate to the position operator $\hat{a}$ (i.e. the momentum). The translation operator effects the following transformation on unnormalised position states of the pointer $\Gamma_{w_A}^{\epsilon}|x_a\rangle = |x_a + \epsilon\rangle$. Analogously we make the same definitions for quantity B. Differences in the average position of the weight before and after the protocol allows us to read off the change in the work of that quantity.

Our intention here is to remain as general as possible, whilst eliminating the possibility of 'cheating' by bringing in resources from outside this framework (such as external sources of work or free energy), or making use of objects within the framework for a purpose other than intended (for example, by using the batteries as a cold reservoirs in generalised heat engines). We make four assumptions on our scheme:

I We assume that the *battery systems are independent* of one another and only accept and store one type of conserved quantity. As such, each quantity is assigned its own battery system.

II The set of allowed operations will consist of *global unitaries* on the bath, system and weights, U, which conserve A and B. Using rule I we have

$$\begin{aligned}[U, A_{\mathrm{b}} + A_{\mathrm{s}} + A_{w_A}] = 0 \\ [U, B_{\mathrm{b}} + B_{\mathrm{s}} + B_{w_B}] = 0\end{aligned} \tag{16}$$

In this way, we impose the first laws of thermodynamics for any initial state:

$$\begin{aligned}\Delta A_{\mathrm{b}} + \Delta A_{\mathrm{s}} + \Delta A_{w_A} = 0 \\ \Delta B_{\mathrm{b}} + \Delta B_{\mathrm{s}} + \Delta B_{w_B} = 0\end{aligned} \tag{17}$$

We choose to study unitaries as opposed to more general completely positive (CP) maps in order not to use external ancillas in non-thermal states as sources of energy or angular momentum.

III We assume *translational invariance* of the weights to reflect the fact that only displacements in the position on the ladders are important. This implies that all unitaries U should commute with translation operators on each weight.

IV Finally, we assume that all four bodies are *initially uncorrelated* and start in the product state $\rho_{\mathrm{s}} \otimes \tau_{\mathrm{b}} \otimes \rho_{w_A} \otimes \rho_{w_B}$.

The proof of the second law in the presence of explicit batteries follows the same logic as the implicit proof up to a few subtleties.

Theorem 1. All unitary evolutions U which are weight-translation invariant cannot decrease the entropy of the system and bath.

$$[U, \Gamma_{w_A}^{\epsilon_1}] = [U, \Gamma_{w_B}^{\epsilon_2}] = 0 \quad \Longrightarrow \quad \Delta S(\rho_{\mathrm{sb}}) \geq 0 \tag{18}$$

where the translation operators Γ are defined in Eq. (15).

Proof. Using the definition in Eq. (15) we associate two momentum-like variables, conjugate to the positions of the pointers, for quantities A and B. For clarity we let $p_a = p$ and $p_b = \varphi$. We argue that since the weights are both translationally invariant, this means that $[U, p] = [U, \varphi] = 0$. Any unitary with this property can be written

$$U = \int dp\, d\varphi\, V(p, \varphi) \otimes |p\rangle\langle p| \otimes |\varphi\rangle\langle\varphi| \tag{19}$$

where the first element of the tensor product $V(p, \varphi)$ corresponds to a unitary operation on the combined system and bath (as a function of the variables conjugate to the positions x_a and x_b of the two weights) and the second corresponds to a projector onto the un-normalised momentum eigenstates $|p\rangle$ and $|\varphi\rangle$ of weights w_A and w_B. By rule IV the weights are initially uncorrelated from the bath and system we can write the initial state (in density matrix form) as $\rho_{\mathrm{sb}} \otimes \rho_{w_A} \otimes \rho_{w_B}$. We are interested in the post-measurement state of ρ'_{sb}. After applying the unitary and tracing out the battery systems, the state is

$$\begin{aligned}\rho'_{\mathrm{sb}} &= \mathrm{tr}_{w_A w_B}\left(U(\rho_{\mathrm{sb}} \otimes \rho_{w_A} \otimes \rho_{w_B})U^\dagger\right) \\ &= \mathrm{tr}_{w_A w_B}\left(\int dp\, dp'\, d\varphi\, d\varphi'\, V(p, \varphi)\rho_{\mathrm{sb}}V^\dagger(p', \varphi') \otimes \left(|p\rangle\langle p|\rho_{w_A}|p'\rangle\langle p'| \otimes |\varphi\rangle\langle\varphi|\rho_{w_B}|\varphi'\rangle\langle\varphi'|\right)\right) \\ &= \int dp\, d\varphi\, V(p, \varphi)\rho_{\mathrm{sb}}V^\dagger(p, \varphi) \otimes \underbrace{\left(\langle p|\rho_{w_A}|p\rangle \otimes \langle\varphi|\rho_{w_B}|\varphi\rangle\right)}_{\text{mixing terms}} \\ &= \int dp\, d\varphi\, V(p, \varphi)\rho_{\mathrm{sb}}V^\dagger(p, \varphi)\, \alpha(p)\nu(\varphi)\end{aligned} \tag{20}$$

where $\alpha(p)$ and $\nu(\varphi)$ are the probability distributions for the momenta on the initial state of the weights. The system and bath therefore evolve via a mixture of unitary transformations. Due to the concavity of the entropy, and the fact that it is preserved under the unitary transformation $V(p, \varphi)$, such evolutions can only increase the entropy of the system and bath

$$\begin{aligned}S(\rho_{\mathrm{sb}}) &\leq S\left(\int dp\, d\varphi\, V(p, \varphi)\rho_{\mathrm{sb}}V(p, \varphi)^\dagger \alpha(p)\nu(\varphi)\right) \\ &= S(\rho'_{\mathrm{sb}}) \\ \Longrightarrow 0 &\leq \Delta S(\rho_{\mathrm{sb}})\end{aligned} \tag{21}$$

We now calculate the von Neumann entropy change of the bath and system. Following rule IV the bath and system are initially uncorrelated, thus their initial entropy is simply the sum of their individual entropies. The unitary we implement may be entangling, and therefore correlations may form between the bath and system during the protocol. Using the result in Eq. (21) and the fact that entropy respects subadditivity, we have that

$$\Delta S_{\mathrm{b}} + \Delta S_{\mathrm{s}} \geq \Delta S_{\mathrm{sb}} \geq 0 \tag{22}$$

$$\Delta S_{\mathrm{b}} \geq -\Delta S_{\mathrm{s}} \tag{23}$$

Following the line of thought from the main text, we now argue that the free entropy of the bath can only increase

$$\Delta \tilde{F}_{\mathrm{b}} = \beta_A \Delta A_{\mathrm{b}} + \beta_B \Delta B_{\mathrm{b}} - \Delta S_{\mathrm{b}} \geq 0. \tag{24}$$

Using rule III and the entropy relation (23) we arrive at the second law

$$\beta_A(-\Delta A_{\mathrm{s}} - \Delta W_A) + \beta_B(-\Delta B_{\mathrm{s}} - \Delta W_B) - \Delta S_{\mathrm{s}} \geq 0 \tag{25}$$

$$\implies \quad \beta_A \Delta \tilde{W}_A + \beta_B \Delta \tilde{W}_B \leq -\Delta \tilde{F}_{\mathrm{s}} \tag{26}$$

Note that if rule II is changed to the case of only average quantity conservation (i.e. Eq. (16)) is dropped) then proof of the second law still holds. Thus our result is universal for both strict and average quantity conservation.

SUPPLEMENTARY NOTE 3

Generalised work extraction details for the case of explicit weights

In this section we show how the protocols for trade-offs between conserved quantities and generalised work extraction in the main paper can be extended from implicit to explicit batteries, in the case of commuting observables. This follows closely the approach in [4, 5].

Any protocol on the system and bath in the implicit battery framework can be represented by a total unitary transformation U (which may be the product of several unitary steps). We can write this transformation as

$$U = \sum_{ij} U_{ij} |i\rangle\langle j| \tag{27}$$

where the basis states $|i\rangle$ are joint eigenstates of $A_{\mathrm{s}} + A_{\mathrm{b}}$ and $B_{\mathrm{s}} + B_{\mathrm{b}}$ with eigenvalues a_i and b_i respectively.

In the explicit battery framework described in the previous section, a general unitary U would not be allowed as it does not strictly conserve the quantities A and B. However, we can instead perform the unitary

$$\tilde{U} = \sum_{ij} U_{ij} |i\rangle\langle j| \otimes \Gamma_{w_A}^{a_j - a_i} \otimes \Gamma_{w_B}^{b_j - b_i}. \tag{28}$$

which commutes with $A_{\mathrm{total}} = A_{\mathrm{s}} + A_{\mathrm{b}} + A_{w_A}$ and $B_{\mathrm{total}} = B_{\mathrm{s}} + B_{\mathrm{b}} + B_{w_A}$. We will now show that for appropriate initial states of the weights (in particular very broad coherent states with momentum approximately zero) $\tilde{U}$ has approximately the same effect on the system and bath as U. Due to the first laws, the work extracted into the weights will then be approximately the same as in the case with implicit batteries. Furthermore, this approximation can be made as good as desired, and the protocol does not degrade the state of the weights for use in further protocols.

In the momentum representation, this can be written

$$\tilde{U} = \int\int V(p,\varphi) \otimes |p\rangle\langle p| \otimes |\varphi\rangle\langle\varphi| dp\, d\varphi \tag{29}$$

where $V(p,\varphi) = \sum_{ij} U_{ij} e^{-ip(a_j - a_i)} e^{-i\varphi(b_j - b_i)} |i\rangle\langle j|$.

Ideally, in order to implement U, we want $V(p,\varphi) = V(0,0) = U$, which corresponds to a very narrow wavefunction for the momentum of w_A and w_B. To show that this can always be done, we trivially extend the proof of Malabarba et al. in Theorem 1 of [4] to the case of two battery systems and show that the state of the system ρ'_{s} after the global unitary will remain close in trace distance to that of the desired local evolution $U\rho_{\mathrm{s}}U^\dagger$. Let $\rho'_{\mathrm{sb}} = \mathrm{tr}_{w_A w_B}(\tilde{U}\rho_{\mathrm{sb}} \otimes \rho_{w_A} \otimes \rho_{w_B} \tilde{U}^\dagger)$, we thus want to show

$$\begin{aligned} &\left\| \mathrm{tr}_{w_A w_B}(\tilde{U}\rho_{\mathrm{sb}} \otimes \rho_{w_A} \otimes \rho_{w_B}\tilde{U}^\dagger) - U\rho_{\mathrm{sb}}U^\dagger \right\| \\ &\qquad = \left\| \int\int dp\; d\varphi \langle p|\rho_{w_A}|p\rangle\langle\varphi|\rho_{w_B}|\varphi\rangle V(p,\varphi)\rho_{\mathrm{sb}}V^\dagger(p,\varphi) - U\rho_{\mathrm{sb}}U^\dagger \right\| \leq \epsilon \end{aligned} \tag{30}$$

for an arbitrary $\epsilon > 0$. $\langle p|\rho_{w_A}|p\rangle$ and $\langle\varphi|\rho_{w_B}|\varphi\rangle$ are well defined probability distributions of the two weights, which we will denote $\mu_{w_A}(p)$ and $\mu_{w_B}(\varphi)$. Since $V(p,\varphi)$ is a continuous function of its variables, then there always exists δ, Δ such that

$$\max_{(p,\varphi)\in I}\left\|V(p,\varphi)\rho_{\rm sb}V^\dagger(p,\varphi) - V(0,0)\rho_{\rm sb}V(0,0)^\dagger\right\| < \frac{\epsilon}{2} \tag{31}$$

where $I = \{(-\delta,\delta),(-\Delta,+\Delta)\}$. We now choose initial weight states such that the probability distributions $\mu_{w_A}(p)$ and $\mu_{w_B}(\varphi)$ satisfy

$$\int_{-\delta}^{\delta}\mu_{w_A}(p)\,dp\int_{-\Delta}^{\Delta}\mu_{w_B}(\varphi)d\varphi \geq 1-\frac{\epsilon}{2}. \tag{32}$$

Substituting Eqs. (31) – (32) into Eq. (30)) one arrives at the result,

$$\left\|\mathrm{tr}_{w_A\,w_B}(\tilde{U}\rho_{\rm sb}\otimes\rho_{w_A}\otimes\rho_{w_B}\tilde{U}^\dagger) - U\rho_{\rm sb}U^\dagger\right\| \leq \epsilon \tag{33}$$

as desired. Note that $\mu_{w_A}(p)$ and $\mu_{w_B}(\varphi)$ are not changed by the protocol, due to the form of Eq. (29), and hence the weights can be reused in future protocols without being degraded.

For the work extraction protocol in particular, the total $\tilde{U}$ will be equal to a product of unitaries for each individual step, $\tilde{U} = \tilde{U}_1\tilde{U}_2$. As an explicit example, the unitary for the first step, in which $\rho_{\rm s}$ is rotated into the joint eigenbasis of A and B is given by

$$\tilde{U}_1 = \sum_{ij} c^*_{ij}|a_i,b_i\rangle\langle a_j,b_j|_{\rm s}\otimes\mathbb{I}_{\rm b}\otimes\Gamma^{a_i-a_j}_{w_A}\otimes\Gamma^{b_i-b_j}_{w_B}, \tag{34}$$

where $|\psi_i\rangle = \sum_j c_{ij}|a_j,b_j\rangle$. The unitary which swaps a two-dimensional subspace of the bath and system is given by

$$\tilde{U}_2 = \mathbb{I}_{\rm sb}\otimes\mathbb{I}_{w_A}\otimes\mathbb{I}_{w_B} + \Bigg(|\mathbf{n}'\alpha',0\rangle\langle\mathbf{n}\alpha,1|_{\rm sb}\otimes\Gamma^{\epsilon_1}_{w_A}\otimes\Gamma^{\epsilon_2}_{w_B} + |\mathbf{n}\alpha,1\rangle\langle\mathbf{n}'\alpha',0|_{\rm sb}\otimes\Gamma^{-\epsilon_1}_{w_A}\otimes\Gamma^{-\epsilon_2}_{w_B}\Bigg) \\ - \Bigg((|\mathbf{n}\alpha,1\rangle\langle\mathbf{n}\alpha,1|_{\rm sb} + |\mathbf{n}'\alpha',0\rangle\langle\mathbf{n}'\alpha',0|_{\rm sb})\otimes\mathbb{I}_{w_A}\otimes\mathbb{I}_{w_B}\Bigg) \tag{35}$$

where $|\mathbf{n}\,\alpha\rangle = |n_0,n_1,n_2,n_3,\cdots n_d,\alpha\rangle$ and $|\mathbf{n}'\alpha'\rangle = |n'_0,n'_1,n'_2,n_3,\cdots n_d,\alpha'\rangle$ are the two states in occupation notation which we choose from the bath. In order to obey strict quantity conservation the weights must shift by the difference in the quantity gap in the system and bath, i.e. $\epsilon_1 = ((a^s_1-a^s_0)-(a^b_{\mathbf{n}'}-a^b_{\mathbf{n}}))$ and similarly $\epsilon_2 = ((b^s_1-b^s_0)-(b^b_{\mathbf{n}'}-b^b_{\mathbf{n}}))$, where a^s_i, b^s_i denote the eigenvalues of $A_{\rm s}, B_{\rm s}$ and a^b_i, b^b_i denote those of $A_{\rm b}, B_{\rm b}$.

SUPPLEMENTARY NOTE 4

A robust protocol in the case of experimental uncertainty

We present a protocol for work extraction from a system with multiple conserved observables in conjunction with a generalised thermal bath, which is robust even in the case that we have uncertainty in the temperatures of the baths. We wish to extract some amount of W_A and W_B and in order to do this we must implement the swap operation in Eq. (35).As such we wish to match the ratio of probabilities $\frac{p'}{p}$ in the system $\rho_{\rm s}$ with the ratio of probabilities $\frac{q'_{\mathbf{n}}}{q_{\mathbf{n}}}$ in the bath $\tau(\beta_A,\beta_B)$.

Specifically, the subscripts $\mathbf{n}$ and $\mathbf{n}'$ in the bath refer to the particularly chosen occupation states (level distributions) $\mathbf{n}$ and $\mathbf{n}'$ of the bath, which we will swap to implement the protocol. $\mathbf{n} = (n_0,n_1,n_2,n_3\cdots,n_d)$ and $\mathbf{n}' = (n'_0,n'_1,n'_2,n_3,\cdots,n_d)$ such that $\mathbf{n}-\mathbf{n}' = ((n_0-n'_0),(n_1-n'_1),(n_2-n'_2),0\cdots,0)$. For commuting observables A, B, the bath probabilities take the following form:

$$q_i \propto e^{-(\beta_A a_i + \beta_B b_i)} \tag{36}$$

where $a_i(b_i)$ are the eigenvalues of the observable $A\,(B)$. Since we have taken the tensor product of n thermal states $\tau(\beta_A, \beta_B)$, the ratio of probabilities between the two selected levels is simply

$$\frac{q_{\mathbf{n}}}{q_{\mathbf{n}'}} = \frac{\prod_{i=0}^{d-1} q_i^n}{\prod_{i=1}^{d-1} q_i^{n_i'}} \tag{37}$$

$$= q_0^{n_0-n_0'} q_1^{n_1-n_1'} q_2^{n_2-n_2'} \tag{38}$$

$$= \left(\frac{q_0}{q_1}\right)^{n_1'-n_1} \left(\frac{q_0}{q_2}\right)^{n_2'-n_2} \tag{39}$$

where we have used particle conservation $\sum_{i=0}^{2} n_i = \sum_{i=0}^{2} n_i'$ to eliminate the term $(n_0 - n_0')$ in the last line. Substituting for the general form of the probabilities from Eq. (36) we have

$$\frac{q_{\mathbf{n}'}}{q_{\mathbf{n}}} = e^{-(x\Delta n_1 + y\Delta n_2)} \tag{40}$$

$$= e^{-y\Delta n_1\left(\frac{x}{y}+\frac{\Delta n_2}{\Delta n_1}\right)} \tag{41}$$

where $x = (\beta_A(a_1 - a_0) + \beta_B(b_1 - b_0))$, $y = (\beta_A(a_2 - a_0) + \beta_B(b_2 - b_0))$ and $\Delta n_1 = (n_1' - n_1)$, $\Delta n_2 = (n_2' - n_2)$. We would like to show that for the quantity in Eq. (40) we can match any possible ratio given to us from the system $\frac{p'}{p}$, which is equivalent to demanding

$$\left| y\Delta n_1 \left(\frac{x}{y} + \frac{\Delta n_2}{\Delta n_1} \right) \right| < \epsilon \tag{42}$$

where ϵ is a constant of $O(\delta p)$.

If we can achieve ϵ sufficiently small, then we can cover $\mathbb{R}^+$ well, in the sense that we can come as close as desired to reproducing any number $0 < \frac{p^0}{p^1} < \infty$ by scaling Δn_1 and Δn_2 by k (where $k \in \mathbb{Z}$).

$$0 < \left((\tfrac{q_{\mathbf{n}'}}{q_{\mathbf{n}}})^k = e^{-yk\Delta n_1(\frac{x}{y}+\frac{\Delta n_2}{\Delta n_1})} > e^{-k\epsilon} \right) < \infty \tag{43}$$

We now present a robust method that will choose Δn_1 and Δn_2 such that Eq.(42) holds, even in the case of uncertainty due to the imprecision of the measuring apparatus.

We begin by making the reasonable assumption that the experimenter measures values which are rational – for instance because the measuring apparatus displays a finite string of decimal digits. The experimenter measures the temperatures of the thermal bath β_A, β_B and computes $\frac{x}{y}$, specifying the uncertainty with δ. They then find $\frac{x}{y} = \frac{u}{v}$ in its reduced form, such that u, v are relative primes. For simplicity, we will consider the case where $0 < x < y$, in which case $0 < \frac{x}{y} < 1$ (the other cases follow similarly).

The exponent we wish to make small becomes

$$\left| y\Delta n_1 \left(\frac{u}{v} + \frac{\Delta n_2}{\Delta n_1} \right) \right| = \left| \frac{y}{v}(u\Delta n_1 + v\Delta n_2) \right| \tag{44}$$

The best we can do is to appeal to number theory: since u an v are relative primes, by Bézout's lemma [6], the smallest (in magnitude) non-zero value of $u\Delta n_1 + v\Delta n_2$ is 1, and there exists a pair $\{\Delta n_1, \Delta n_2\}$ for which this is true. For this choice of pair, the quantity in Eq. (44) is automatically less than ϵ if $\frac{y}{v} < \epsilon$. What we find is that in the case that $\frac{y}{v} > \epsilon$, we do not know how to minimise this quantity because our best method fails. Thus, for fixed ϵ, there is a finite set of points $\{\frac{p}{q}\}^F$ that are excluded from our protocol. This set is precisely the Farey sequence of order $\lfloor \frac{y}{\epsilon} \rfloor$, (where $\lfloor \bullet \rfloor$ denotes the floor function: the largest integer not greater than $\bullet$).

Definition 1 (Farey Sequence). "A Farey sequence of order n is the ascending series of irreducible fractions between 0 and 1 whose denominators do not exceed n". [7]. For example the sequence of order 5 is $F_5 = \{\frac{0}{1}, \frac{1}{4}, \frac{1}{3}, \frac{1}{2}, \frac{2}{3}, \frac{3}{4}, \frac{4}{5}, \frac{1}{1}\}$.

We proceed by constructing a robust protocol around these excluded points. The experimenter measures $\frac{x}{y}$, computes $\frac{u}{v}$ and identifies the closest rational $\frac{u^*}{v^*}$ from the Farey sequence of order $\lfloor \frac{y}{\epsilon} \rfloor$ to the computed point. It then suffices to choose $\{\Delta n_1 = u^*, \Delta n_2 = -v^*\}$. With this choice

$$|x\Delta n_1 + y\Delta n_2| = \left| yv^* \left(\frac{x}{y} - \frac{u^*}{v^*} \right) \right| \tag{45}$$

$$< \epsilon \quad \textit{iff} \quad \left| \frac{x}{y} - \frac{u^*}{v^*} \right| < \frac{\epsilon}{yv^*} \tag{46}$$

For this choice, the quantity $|x\Delta n_1 + y\Delta n_2| \le \epsilon$ for the interval $I_{u^*/v^*} = \left(\frac{u^*}{v^*} - \frac{\epsilon}{yv^*}, \frac{u^*}{v^*} + \frac{\epsilon}{yv^*}\right) - \{\frac{u^*}{v^*}\}$.

This method will work for the case that all the intervals overlap, otherwise there would be regions for which the protocol didn't work. The fact that the intervals overlap is the subject of the next theorem.

Theorem 2 (Farey intervals overlap). The union of all intervals around each member of the Farey sequence of order $\lfloor \frac{y}{\epsilon} \rfloor$ cover the unit interval on which the Farey sequence is defined.

$$\bigcup_i I_{u/v_i} > [0,1] \tag{47}$$

where u/v_i is the i-th element of the Farey sequence and $I_{u/v} = \left(\frac{u}{v} - \frac{\epsilon}{yv}, \frac{u}{v} + \frac{\epsilon}{yv}\right) - \{\frac{u}{v}\}$ is the interval.

Proof. To prove that the collection of intervals around all bad rationals $\frac{u}{v}$ covers the real line, it suffices to prove that the neighbouring intervals in this collection intersect. Consider the rational number $\frac{u'}{v'}$ that is the next rational number (i.e. the neighbour) of $\frac{u}{v}$ in the Farey sequence of order $\lfloor \frac{y}{\epsilon} \rfloor$. Then the corresponding interval is $I_{u'/v'} = \left(\frac{u'}{v'} - \frac{\epsilon}{yv'}, \frac{u'}{v'} + \frac{\epsilon}{yv'}\right) - \{\frac{u'}{v'}\}$. Comparing the supremum of the interval about $\frac{u}{v}$ and the infimum of the interval about $\frac{u'}{v'}$, and using the properties of neighbours in a Farey sequence, one has that

$$\sup\,\left(I_{u'/v'}\right) - \inf\,\left(I_{u/v}\right) = \frac{u'}{v'} - \frac{u}{v} - \frac{\epsilon}{y}\left(\frac{1}{v'} + \frac{1}{v}\right) \tag{48}$$

$$= \frac{1}{vv'} - \frac{\frac{\epsilon}{y}(v+v')}{vv'}, \tag{49}$$

where we have used the property that if $\frac{u}{v}$ and $\frac{u'}{v'}$ are neighbours in a Farey sequence, then $\frac{u'}{v'} - \frac{u}{v} = \frac{1}{vv'}$ [7].

Furthermore, if $\frac{u}{v}$ and $\frac{u'}{v'}$ are neighbours in a Farey sequence of order $\lfloor \frac{y}{\epsilon} \rfloor$, then $v + v' > \frac{y}{\epsilon}$, else the mediant $\frac{(u+u')}{(v+v')}$ would also be in the Farey sequence of order $\lfloor \frac{y}{\epsilon} \rfloor$ which contradicts the assumption that $\frac{u}{v}$ and $\frac{u'}{v'}$ are neighbours. Therefore it follows from Eq. (49) that $\sup\,\left(I_{u'/v'}\right) - \inf\,\left(I_{u/v}\right) < 0$. Thus the intervals overlap, and the union of the intervals around every rational number in the sequence covers the real line (less the excluded rational numbers) $\square$.

Finally, the experimenter must verify that the uncertainty in their measurement, δ, falls within the range of the Farey interval they have chosen, $\frac{x}{y} \pm \delta \in I_{u^*/v^*}$. If this criterion is not met, then the experimenter is obliged to respecify either ϵ or δ, i.e. to respecify the fine-graining of the bath or make a more accurate measurement. These arguments are easy to extend to the real line: the Farey sequence is translationally invariant on any unit interval and can be scaled by a constant as necessary; the experimenter constructs the sequence of order $\lfloor \frac{y}{\epsilon} \rfloor$ between the integers that $\frac{x}{y}$ lies in, i.e. in the interval $\left\lfloor \frac{x}{y} \right\rfloor < \frac{x}{y} < \left\lceil \frac{x}{y} \right\rceil$, (where $\lceil \bullet \rceil$ denotes the ceiling function: the smallest integer not less than $\bullet$). The full protocol is summarised in Supplementary Fig. 3

SUPPLEMENTARY REFERENCES

[1] A. Hatcher, Topology of numbers, http://www.math.cornell.edu/ hatcher/TN/TNpage.html, (2002).
[2] E. T. Jaynes, *Information Theory and Statistical Mechanics*, Phys. Rev. **106**, 620–630 (1957).
[3] Y.-K. Liu, *Gibbs States and the Consistency of Local Density Matrices*, Preprint at http://arxiv.org/abs/quant-ph/0603012 (2006).
[4] A. S. L. Malabarba, A. J. Short, and P. Kammerlander, *Clock-driven quantum thermal engines*, New J. Phys. **17**, 045027 (2015).
[5] J. Åberg, *Catalytic Coherence*, Phys. Rev. Lett. **113**, 150402 (2014).
[6] G. Andrews, *Number Theory*, Dover Books on Mathematics (Dover Publications, 1971).
[7] G. Hardy and E. Wright, *An Introduction to the Theory of Numbers*, Oxford science publications (Clarendon Press, 1979).
